# Fufang shenhua tablet, astragali radix and its active component astragaloside IV: Research progress on anti-inflammatory and immunomodulatory mechanisms in the kidney

**DOI:** 10.3389/fphar.2023.1131635

**Published:** 2023-04-05

**Authors:** Run Li, Chunru Shi, Cuiting Wei, Chao Wang, Hongjian Du, Quan Hong, Xiangmei Chen

**Affiliations:** ^1^ The College of Traditional Chinese Medicine, Guangdong Pharmaceutical University, Guangzhou, China; ^2^ Department of Nephrology, First Medical Center of Chinese PLA General Hospital, Nephrology Institute of the Chinese People’s Liberation Army, State Key Laboratory of Kidney Diseases, National Clinical Research Center for Kidney Diseases, Beijing Key Laboratory of Kidney Disease Research, Beijing, China

**Keywords:** Fufang Shenhua Tablet, Astragali Radix, Astragaloside IV (AS-IV), anti-inflammatory, immunity, kidney disease

## Abstract

**Background:** Given the limited treatment options available for kidney disease, a significant number of patients turn to alternative therapies, including traditional Chinese medicine. Among these therapies, the Fufang Shenhua tablet (SHT) has garnered attention for its effectiveness in addressing the most common deficiency of Qi and Yin in chronic glomerulonephritis. Notably, the sovereign drug of SHT is Astragali Radix (AR), with the most abundant and effective component being Astragaloside IV (AS-IV). AS-IV has been shown to possess anti-inflammatory and immunomodulatory properties, and it is extensively used in treating kidney diseases. Nevertheless, the molecular mechanisms underlying its action are numerous and intricate, and a comprehensive understanding is yet to be achieved.

**Aim of the review:** Thus, we have thoroughly examined the existing research and outlined the advancements made in investigating the anti-inflammatory and immunomodulatory mechanisms of SHT, AR and its active component AS-IV, in relation to kidney health. This serves as a dependable foundation for conducting more comprehensive investigations, evaluating efficacy, and making further improvements in the future.

**Materials and methods:** We conducted a comprehensive literature search utilizing multiple globally recognized databases, including Web of Science, Google Scholar, PubMed, ScienceDirect, Wiley, ACS, Springer, and CNKI. The search keywords used in this study were “Fufang Shenhua tablet,” “Astragali Radix,” “Astragaloside IV,” and “Anti-inflammatory” or “Immunity.”

**Results:** The mechanism of inflammation inhibition by SHT, AR and its active component AS-IV is mainly related to the signaling pathways such as NF-κB, TLRs, PI3K/AKT, Wnt/β-catenin, and JAK-STAT. Immunomodulation exerts not only activating, stimulating, and regulating effects on macrophages and dendritic cells, but also on immune organs, T-lymphocytes, B-lymphocytes, and a myriad of cytokines. Moreover, the SHT, AR and its active component AS-IV also demonstrate regulatory effects on renal cells, including glomerular mesangial cells, tubular epithelial cells, and podocytes.

**Conclusion:** To summarize, SHT, AR and its active component AS-IV, exhibit notable therapeutic effects in kidney-related ailments, and their molecular mechanisms for anti-inflammatory and immunomodulatory effects have been extensively explored. However, further standard clinical trials are necessary to evaluate their safety and efficacy in the adjunctive treatment of kidney-related diseases. Moreover, in-depth studies of unverified chemical components and regulatory mechanisms in SHT are required. It is our belief that with continued research, SHT, AR and its active component AS-IV are poised to pave the way for enhancing therapeutic outcomes in kidney-related ailments.

## 1 Introduction

Over the last two decades, close to 850 million individuals have been afflicted by various forms of kidney ailments (Basso et al., 2021). Due to the lack of specific therapeutic drugs, many of these conditions progress to end-stage renal disease (ESRD). At present, renal replacement therapy remains the only effective treatment for ESRD, but it places a significant economic burden on healthcare systems and individuals. Hence, there is an urgent need to investigate potential alternative treatments, such as traditional Chinese medicine (TCM), and develop sustainable and effective strategies to prevent ESRD.

Fufang Shenhua tablet (SHT) is a TCM compound developed under the guidance of TCM theory and long-term clinical experience. SHT is composed of seven TCMs (Table1): *Astragalus Radix* (AR), *Atractylodis Macrocephalae Rhizoma*, *Ligustri Lucidi Fructus*, *Paeoniae Radix Alba*, *Sparganii Rhizoma*, *Curcumae Rhizoma*, and *Lonicerae Japonicae flos*. AR is the sovereign drug, *Ligustri Lucidi fructus,* and *Curcumae Rhizoma* are minister drugs, and *Atractylodis Macrocephalae Rhizoma, Paeoniae Radix Alba, Sparganii Rhizoma* and *Lonicerae Japonicae flos* are assistant drug, and its main functions are supplementing Qi, nourishing Yin, promoting blood circulation, removing blood stasis, and clearing away heat. The therapeutic indications are to treat chronic nephritis, proteinuria, or with early to mid-stage renal insufficiency, accompanied by TCM symptoms of Qi and Yin deficiency, and mutual stasis and toxicity. Following a preliminary clinical study, SHT was observed to significantly decrease the Chinese medicine symptom scores of Qi and Yin deficiency in patients suffering from chronic nephritis. Moreover, it was also found to significantly reduce urine protein levels, with a notable effect in halting the progression of nephritis (Chen). In a subsequent clinical study, a prospective, multicenter, double-blind, double-modeled, randomized controlled trial design protocol was utilized to compare SHT with the angiotensin-converting enzyme inhibitor fosinopril, which was used as a positive control drug. The results of the study indicated that after 12 weeks of treatment, SHT and fosinopril exhibited similar efficacy in reducing urinary protein levels in patients displaying Qi and Yin deficiency evidence of IgA nephropathy, without any severe adverse effects (Chen et al., 2007). Moreover, animal experiments have also corroborated the therapeutic effect of SHT in treating chronic anti-Thy-1 tract proliferative glomerulonephritis, diabetic nephropathy, and other chronic kidney diseases (Geng et al., 2012; Geng et al., 2016).

**TABLE 1:** Composition of SHT.

Herbal name in Chinese	Herbal composition	Plant scientific name	Part used	Weight ratio
Huang Qi	Astragalus Radix	*Astragalus membranaceus (Fisch.) Bge.var.mongholicus* (*Bge.*)*Hsiao or Astragalus membranaceus* (*Fisch.*)*Bge*	Radix	5
Bai Zhu	Atractylodis Macrocephalae Rhizoma	*Atractylodes macrocephala Koidz*	Radix or Rhizoma	3
Nv Zhen Zi	Ligustri Lucidi Fructus	*Ligustrum lucidum Ait*	Fructus	3
Bai Shao	Paeoniae Radix Alba	*Paeonia lactiflora Pall*	Radix	3
San Leng	Sparganii Rhizoma	*Sparganium stoloniferum Buch.-Ham*	Rhizoma	4
E Zhu	Curcumae Rhizoma	*Curcuma phaeocaulis VaL. or Curcuma kwangsiensis S.G.Lee et C.F.Liang*	Radix or Rhizoma	4
Jin Yin Hua	Lonicerae Japonicae flos	*Lonicera japonica* Thunb	Flos	4

**FIGURE 1:**
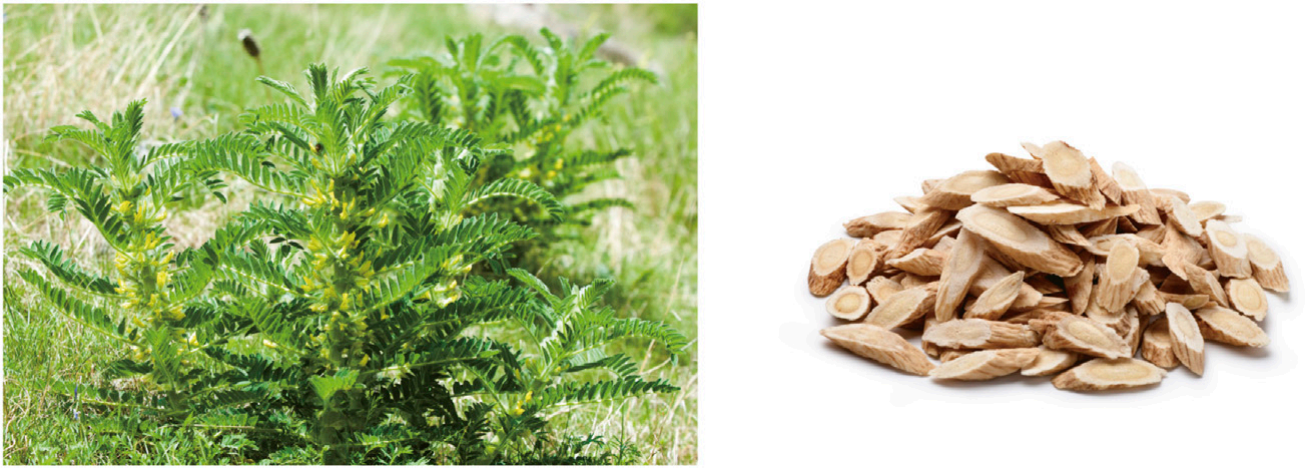
Astragali Radix herbs and slices.

Astragali Radix (AR, Huangqi in Chinese, also known as astragalus), which is the sovereign drug of SHT, is the dried root of *Astragalus membranaceus* (Fisch.) Bge. var. *mongholicus* (Bge.) Hsiao or A. *membranaceus* (Fisch.) Bge. (Figure 1). AR has a rich history of over 2,000 years of use in China and is widely used in clinical practice. Its sweet and mild nature characterizes it, and it is associated with the meridians of the lung and spleen. This herb is classified as a qi-tonifying medicine (Commission, 2020). As one of the TCM frequently employed in clinical practice, AR exhibits a wide array of pharmacological effects, such as antioxidation (Shahrajabian et al., 2019), immunomodulation (Block and Mead, 2003), antifibrosis (Zhou et al., 2016), antitumor (Zhou et al., 2018), and analgesic effects (Di Cesare Mannelli et al., 2017). Like most botanical remedies, AR comprises a multitude of natural constituents with distinct structural characteristics. AR is rich in flavonoids, saponins, polysaccharides, amino acids, and numerous other compounds that exhibit diverse biological activities both *in vivo* and *in vitro*. However, not all components present in AR possess immune-inflammatory modulation capabilities. Relevant research suggests that the primary active ingredients responsible for immune-inflammatory modulation in AR are Astragalus polysaccharides (APS), Astragalus saponins (AS), and Astragalus flavonoids (AF) (Li et al., 2009).

**FIGURE 2:**
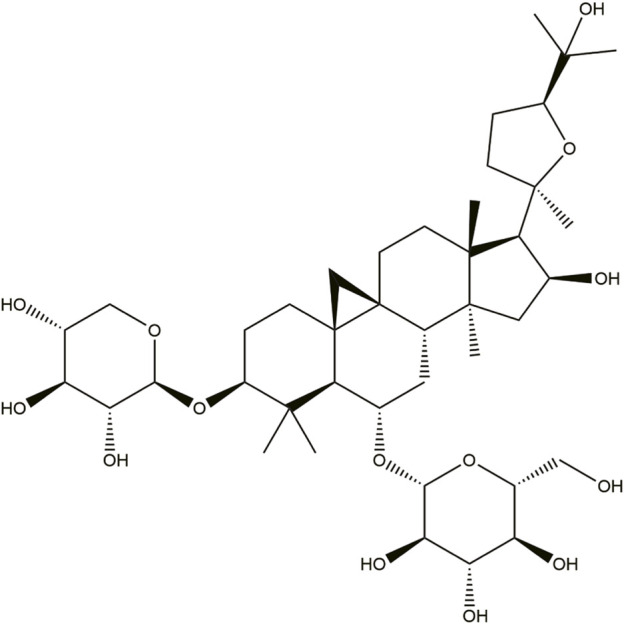
Chemical structure of Astragaloside IV.

Astragaloside IV (AS-IV) is a primary active component found in AR and is utilized as a quality control marker of Huangqi as per the Chinese Pharmacopeia (2015 version). AS-IV can be obtained and separated using various methods, including ultrafiltration, high-speed centrifugation, reflux, water extraction, ultrasonic extraction, and ethanol precipitation (Ren et al., 2013). AS-IV is a cyclopropane triterpene saponin with a molecular formula of C_14_H_68_O_14_ and a relative molecular mass of 784.97 (Figure 2). It exhibits a diverse range of pharmacological activities, including antioxidant, cardioprotective, anti-inflammatory, antiviral, antibacterial, antifibrotic, anti-diabetic, and immunoregulatory effects. These properties make it a promising therapeutic candidate for treating cerebral injury and central nervous system, cardiovascular disease, respiratory system, kidney, endocrine system, organic immune system, liver, and cancer (Ren et al., 2013; Li et al., 2017).

In this manuscript, we appraise the involvement of SHT and AR, alongside their chief activity AS-IV, in the orchestration of immune-inflammatory responses, delineating their mechanisms of action and therapeutic applications in the management of renal disorders. Our objective is to establish a theoretical framework for their subsequent pharmacological scrutiny and clinical implementation.

## 2 Anti-inflammation

### 2.1 Nuclear factor kappa-light-chain-enhancer of activated B cells (NF-κB) signaling pathways

NF-κB, which interacts closely with inhibitory proteins (IκB) and IκB kinase (IKK), is a key intracellular molecule that mediates the inflammatory response. IκB and IKK are two essential upstream modulatory elements of the NF-κB signal transduction cascade. IKK-β is a protein subunit of IκB-α that can bind to cytoplasmic NF-κB and keep it inactive. After translocating into the nucleus, NF-κB is activated and promotes the transcription of proinflammatory factors such as TNF-α, TNF-α, IL-1β, IL-6 and MCP-1 (Hong et al., 2017).

In a rat model of IgA nephropathy induced by bovine serum albumin (BSA) gavage, LPS, and CC14 injection, the oral administration of AR (3 g/kg/d for 6 weeks) was found to significantly reduce urinary erythrocyte count, urinary protein, and β-N-acetylaminoglucosidase (NAG) content, and also alleviate renal tubulointerstitial lesions in IgAN rats. These beneficial effects are thought to be attributed to the downregulation of NF-κB and MCP-1 expression (Zhang et al., 2008). Moreover, AR injection (2, 20, 200 μg/L for 48 h, 72 h) was shown to inhibit high glucose-induced apoptosis and NF-κB protein expression in renal tubular epithelial cells (HK-2). The inhibitory effect was found to be positively correlated with the dose. Further investigations revealed that intraperitoneal injection of AR injection (1 mL/week for 7 weeks) reduced the expression rate of NF-κB and cytokine TNF in rats with immune complex nephritis model established by tail vein injection with BSA. This suggests that AR injection may exert therapeutic effects by suppressing the immune response through the reduction of NF-κB expression (Wang et al., 2001; Mei et al., 2016). Additionally, in the lipopolysaccharide (LPS)-induced diabetic nephropathy (DN) rat model, AR decoction (1.5, 3.0 g/kg for 8 weeks) was found to significantly improve general indices, renal function, and proteinuria. It was also observed to decrease the phosphorylation of IKK and NF-κB by reducing the expression of TLR4, thus reducing downstream inflammatory factors such as IL-6, TNF-α, and IL-1β expression levels. These results suggest that AR decoction may exert an inhibitory effect on TLR4/NF-κB signaling pathway, thereby reducing the inflammatory response and improving renal pathological indices (Li, 2019).

### 2.2 Toll-like receptors (TLRs) signaling pathways

TLRs are a family of germline-encoded receptors that are responsible for the development of inflammatory and immune responses. TLRs are expressed on various cells, including antigen-presenting cells and intrinsic kidney cells. The recognition of TLR ligands prompts the innate immune response and stimulates TLR signaling, which initiates M1 macrophage polarization and infiltration and mediates the transcription of NF-κB, which is followed by the inflammatory cascade and proinflammatory cytokine and chemokine release. Notably, almost all TLRs (except for TLR3) use myeloid differentiation primary response 88 (MYD88) as a general adapter protein to activate NF-κB. Activation of the TLR signaling pathway has been reported to exacerbate inflammation (Zhang T. et al., 2017; Zhao and Han, 2018).

Administration of SHT (1.5, 3.0 g/kg for 7 days prior to pre-operation) significantly decreased the protein expression and RNA levels of TLR2 and TLR4 in rats with renal ischemia-reperfusion injury (IRI), and its improvement of AKI symptoms may be achieved by reducing the expression of these renal immune inflammatory receptors (Zheng et al., 2012). The effects of SHT on inflammatory cytokines associated with TLR2 and TLR4, as well as MyD88, were further investigated. The results showed that SHT intervention significantly reduced the levels of IL-6, IL-12, and MyD88 protein and mRNA levels in the kidneys of rats, and also led to a decrease in serum levels of IL-8 and INF-γ (Li et al., 2019). In another study, AS-IV (20, 40 mg/kg for 1 week) was found to significantly reduce fibronectin (FN) and collagen I (COL-1) levels, as well as inhibit inflammatory cell infiltration and cytokine secretion in a mouse model of UUO-induced renal fibrosis. *In vitro* experiments with human proximal tubular epithelial cells (HK-2 cells) stimulated with LPS (100 ng/mL) also demonstrated that AS-IV (10, 20 μM for 48 h) inhibited TNF-α and IL-1β expression, and these results suggest that AS-IV may protect against the progression of renal fibrosis by suppressing inflammation through the TLR4/NF-κB signaling pathway. Furthermore, AS-IV was found to decrease NF-κB expression and increase IκBα expression (Zhou et al., 2017).

### 2.3 PI3K/AKT signaling pathways

The PI3K/AKT signaling pathway is vital for cell proliferation, growth and viability. As an upstream modulator of NF-κB, PI3K/AKT signaling pathway activation has been demonstrated to ameliorate inflammation (Hong et al., 2017).

Treatment with AR (5 g/kg for 8 weeks) resulted in a reduction of 24-hour urinary protein levels in DN rats, with its efficacy becoming progressively evident as the duration of treatment was extended. The underlying mechanism may involve the reduction of expression levels of p-PI3K, p-Akt, NF-κB, MCP-1, and mRNA to regulate the PI3K/Akt/NF-κB signaling pathway in DN rats, thereby attenuating renal injury (Shen, 2016). In a similar vein, treatment with AS-IV (20, 40, 80 mg/kg for 8 weeks) in a rat model of type 2 diabetes, established through intraperitoneal injection of streptozotocin (STZ) (35 mg/kg), resulted in improvements in body mass, renal index, blood glucose, 24-hour urine protein, urine microalbumin, urine creatinine, blood creatinine, and blood urea nitrogen levels when compared to the model group. Moreover, renal tissue lesions showed significant improvements, and medium and high doses of AS-IV inhibited expression of p-PI3K/PI3K, p-Akt/Akt, and p-FoxO1/FoxO1. AS-IV also upregulated the expression of BNIP3, LC3-II/LC3-I, and Beclin1, which may offer new perspectives for the clinical treatment of diabetic nephropathy by inhibiting the PI3K/Akt pathway (Ma et al., 2019).

### 2.4 Wnt/β-catenin signaling pathways

The Wnt signaling pathway is controlled by Wnt, which is highly conserved in cells and is involved in many biological processes. To maintain the balance of transduction pathways, Wnt can act through the signaling cascade, and control cell differentiation, migration, apoptosis, and cancerous changes to control the growth and development, disease, and death (Long et al., 2010). Wnt proteins maintain a state of dynamic balance. Dysregulated expression of signaling pathway proteins can lead to various diseases, such as inflammation, carcinogenesis, neurological diseases, respiratory diseases, and metabolic disorders (Koziński and Dobrzyń, 2013).

Wang et al. discovered that overexpression of miR-21 causes the activation of protein expression of β-catenin, which, in turn, promotes renal cell fibrosis through the TGF-β1/Smad pathway in diabetic mice. AS-IV has a therapeutic effect because it downregulates the expression of miR-21 in renal cells. However, the therapeutic effect of AS-IV is suppressed when either the Wnt/β-catenin pathway or TGF-β1/Smad pathway is inhibited. Thus, AS-IV is considered a promising drug for the treatment of glomerular diseases (Wang et al., 2018). Furthermore, Li et al. demonstrated that the Wnt/β-catenin signaling pathway was abnormally activated in the kidneys of UUO rats. AS-IV (3.3, 10, 33 mg/kg for 14 days prior to the operation) inhibited this effect in a concentration-dependent manner and suppressed renal interstitial fibrosis (Wang et al., 2014a). Other studies have also found that AS-IV and APS can decrease the expression of inflammatory factors, such as IL-6, IL-8, and TNF-α, oxidative factors like ROS and MDA, and the apoptotic factor caspase-3 in inflammatory cells. Furthermore, the expression of β-catenin is activated in inflammatory cells for cytoprotection (Fan, 2021). AR treatment (2.1 g/kg for 12 weeks) in STZ-induced DN rats can reduce 24-hour urine protein quantification, attenuate interstitial pathological damage, and inhibit the expression of wnt4, β-catenin, and TGF-β1 in the renal interstitium, thus playing a role in kidney protection and slowing down the process of interstitial fibrosis in DN rats (Deng and Fang, 2012).

### 2.5 JAK-STAT signaling pathways

The JAK-STAT signaling pathway comprises three primary components, including tyrosine kinase-related receptors, the tyrosine kinase JAK, and the transcription factor STAT (Banerjee et al., 2017). In mammals, the JAK family constitutes a non-receptor class of tyrosine kinases that consists of JAK1, JAK2, JAK3, and TYK2. Among these, JAK1, JAK2, and TYK2 are broadly expressed in various tissues and cells, while JAK3 is restricted to bone marrow cells and lymphocytes (Chuang and He, 2010). STATs are a group of cytoplasmic proteins that interact with the regulatory regions of target genes, located downstream of JAKs, and crucially participate in signal transduction and transcriptional activation. The STAT proteins comprise STAT1, STAT2, STAT3, STAT4, STAT5a, STAT5b, and STAT6. JAK-STAT signal transduction and transcriptional activation are the primary regulatory pathways associated with cytokines. Specifically, JAK binds to the intracellular domain of the activated cytokine receptor and transmits the signal to the promoter of the target gene upon activation (Xin et al., 2019).

The immune complex deposition-induced activation of the inflammatory response cascade is a major contributor to kidney injury, with inflammatory cytokines playing a crucial role in the development of chronic nephritis. SHT contains components with immune-enhancing and anti-inflammatory properties, and intervention with SHT (45 g/kg for 4 weeks) was found to decrease the expression and activation levels of STAT3 in the kidney tissue of rats with chronic serum disease nephritis, consequently reducing downstream TIMP-1 expression and inhibiting thylakoid cell proliferation. These findings suggest that SHT have a potential to alleviate renal injury in rats with nephritis (Zhang et al., 2005). Furthermore, in high glucose-treated renal tubular epithelial cells, APS (200 mg/L for 48 h) was shown to effectively inhibit apoptosis, transdifferentiation, and reactive oxygen levels. Further analysis revealed that APS treatment resulted in the downregulation of E-cadherin, α-SMA, p-STAT1, and p-STAT3 protein expressions in renal tubular epithelial cells, indicating that APS may exert its protective effect on renal tubular epithelial cells by regulating the JAK-STAT signaling pathway (Guo et al., 2018).

## 3 Immune regulation

### 3.1 Targeting innate immune cells

Innate immune cells are a vital constituent of the innate immune system, which promptly reacts to invading pathogens either after or concurrently with the fortification of tissue barriers. These cells generate non-specific anti-infective immunity, consisting of neutrophils, macrophages, and natural killer (NK) cells.

#### 3.1.1 Regulation of macrophages

Macrophages are a pivotal innate immune cell type, with an important role in counteracting tumor progression and microbial infection. Macrophage migration inhibitory factor (MIF) is produced by lymphocytes and macrophages, and its concentration is closely associated with the degree of renal histopathological damage. Following administration of SHT (5 g/kg for 12 weeks), inflammatory infiltration and cell proliferation were reduced in renal tissue, along with a decline in the expression of monocyte macrophage antigen (ED-1) due to MIF downregulation. Furthermore, SHT treatment significantly reduced the number of PCNA-positive cells, and decreased the secretion of extracellular matrix (Li, 2005). Upon application of SHT-containing serum to LPS-stimulated renal tubular epithelial cells, the mRNA and protein expression of ICAM-1, VCAM-1, MCP-1, and MIF were significantly reduced, indicating that SHT could suppress immune and inflammatory responses, thus hindering cell proliferation and extracellular matrix synthesis (Li et al., 2005). Moreover, *in vitro* experiments have demonstrated that APS (50, 100 μg/mL for 12 h) enhances macrophage proliferation and boosts the expression of CD80, CD86, and MHC-II on the cell surface, thereby increasing the phagocytosis rate and phagocytic capacity of individual cells (Luo et al., 2022). Non-etheless, the molecular mechanism underlying APS-induced macrophage activation warrants further investigation.

#### 3.1.2 Regulation of NK cells

NK cells are non-specific effector cells that can kill target cells without the need for antibodies or complement, and are particularly effective against a wide range of tumor cells. Xu et al. discovered that high doses of AR particles can affect T lymphocyte subsets, NK cells, and serum platelet-activating factor (PAF) levels in patients, thereby improving their quality of life (Xu et al., 2022). Additionally, AS-IV (50 mg/kg for 28 days) enhances the killing capacity of cyclophosphamide-induced immunosuppressed mice against NK cells, promotes the secretion of IL-2 and IFN-γ, and thus improves immune function in mice (Hu et al., 2021).

#### 3.1.3 Regulation of dendritic cells (DCs)

DCs are specialized antigen-presenting cells with potent immune function *in vivo*. They possess the ability to activate naive T cells and therefore play a critical role in initiating specific immune responses (Sun, 2011).

In their research, Ruan et al. discovered that is, o-AS-IV (10, 20, and 40 mg/kg for 7 days) ameliorated dysregulation of T and B lymphocytes, enhanced lymphocyte proliferation and proliferation index (SI), improved macrophage phagocytosis and IL-6 secretion, while decreasing the proportion of CD3^+^ cells and increasing the proportion of CD19^+^ cells. Furthermore, iso-AS-IV induced the secretion of IL-6 and IL-12p70 by DCs, promoted DC maturation and activation, and increased the expression of CD80, CD86, and MHCII (Ruan et al., 2021). Studies have shown that AS-IV enhances non-specific phagocytosis, antigen presentation, and lymphocyte proliferation, and augments the secretion of DCs with immunostimulatory factors such as IL-1β, IL-6, and IL-12. Additionally, AS-IV further amplifies Th1-mediated immune responses by augmenting the expression of the Th1 cell polarizing cytokine, IL-12. These findings imply that AS-IV plays an important role in immunomodulation (Jeon et al., 2018). Moreover, treatment with APS solution (125 μg/mL for 48 h) promotes the proliferation of bone marrow-derived DCs, enhances the capacity of T cells to proliferate and present antigens, increases the production of interferon-γ and IL-2, and upregulates the expression of CD80 and CD86 (Zhang W. et al., 2017).

### 3.2 Effects on the adaptive immune system and its responses

Adaptive immunity encompasses a complex system of immune organs and specialized immune cells, representing the final and most rigorous line of immune defense.

#### 3.2.1 Regulation of immune organs

Immune organs are specialized tissues and structures where immune cells are produced, differentiated, and integrated. These vital structures facilitate immune function and actively participate in immune responses. Immune organs can be broadly classified into central immune organs, which include the thymus, bone marrow, and bursa of Fabricius, and peripheral immune organs, which include the spleen, lymph nodes, and mucosa-associated lymphoid tissue. Research has shown that AR aqueous decoction had a significant impact on increasing the spleen and thymus indices in rats with spleen deficiency (Zhang et al., 2021). Subsequent experiments demonstrated that AR injections (2.5, 5, 10 mL/kg for 6 days) effectively reduced thymocyte apoptosis in rats with severe pneumonia. This may be attributed to the promotion of B lymphocytoma-2 (Bcl-2) expression, the decrease of Bax expression, and the increase of the bcl-2/Bax ratio (Lei et al., 2011).

#### 3.2.2 Regulation of specific immune cells

B lymphocytes are primarily responsible for producing antibodies and contributing to humoral immunity. T lymphocytes and helper T lymphocytes, also known as Th cells, are able to promote the growth and differentiation of B lymphocytes, while also secreting cytokines to activate macrophages and participate in cell-mediated immune responses. These cells have a range of effects on specific immune cells, including increasing the proliferation and differentiation of B and T lymphocytes, regulating the balance of T lymphocyte subsets, increasing serum antibody concentrations, and enhancing the secretion of plasma cells.

T lymphocytes are crucial cells in adaptive immunity, consisting of two subpopulations: CD4^+^ and CD8^+^, each with distinct functions (Salgame et al., 1991). CD4^+^ cells, also known as helper T cells, play a vital role in initiating and promoting immune responses, while CD8^+^ cells have a suppressive role. Therefore, it is essential to maintain the balance between CD4^+^ and CD8^+^ cells to preserve the normal state of the immune system (Jackutė et al., 2015; Song et al., 2015). *In vitro* studies have shown that APS liposomes (62.5–7.813 μg/mL) can significantly increase B lymphocyte proliferation (Fan et al., 2012). APS interacts with immunoglobulins (Ig) located on the surface of B cells, stimulating B cell proliferation (Shao et al., 2004). Additionally, the AR extract, containing 26% extract, including 200 mg/kg, significantly enhances B cell proliferation in mice (Hong et al., 2018). *In vitro*, AR injection (200,1000 mg/mL) can reduce the level of abnormal O-glycosylation of IgA1 and upregulate the expression of specific molecular chaperones to core Iβ3-Gal-T (Cosmc) in peripheral B lymphocytes. This mechanism is important in the treatment of immunoglobulin A nephropathy (IgAN) (Ji et al., 2014). APS induces differentiation of T cells by inducing and activating T cells to regulate immunity (Liu et al., 2011). Furthermore, the administration of APS (5–20 mg/kg) has been shown to increase the number of CD3^+^CD4^+^CD8^+^ memory T helper (Th) and CD3^+^CD4^−^CD8^+^ cytotoxic T cells in peripheral blood lymphocytes (PBL) of 4-week-old Yorkshire pigs (Li et al., 2011).

## 4 Targeting renal cells

### 4.1 Glomerular mesangial cells

Glomerular mesangial cells, which are intrinsic cells located in the glomerulus between capillary loops, are adjacent to endothelial cells or the basement membrane and exhibit an irregular shape. Their cell processes can be deeply located between endothelial cells and the basement membrane or can extend through endothelial cells into the capillary lumen. Glomerular mesangial cells perform various functions such as the secretion of extracellular matrix, production of cytokines, support of the glomerular capillary plexus, phagocytosis and removal of macromolecular substances, and contraction similar to smooth muscle cells.

Interstitial proliferative nephritis is a prevalent form of chronic glomerulonephritis. Research has indicated that after a duration of 3 months of administering SHT (0.75, 1.5, 3 g/kg), the levels of urine protein, serum creatinine, and total cholesterol showed a significant decrease. Furthermore, serum total protein showed an increase, while the number of PCNA-positive cells in interstitial cells decreased significantly in rats. These findings indicate that SHT is effective in ameliorating the symptoms of Thy1 nephritis and inhibiting the proliferation of interstitial cells in rats (Geng et al., 2016). Additionally, Wang et al. discovered that AS-IV increased the expression of CHOP, BAX, and cleaved caspase3, which regulate apoptosis, by upregulating the phosphorylation of endoplasmic reticulum stress-related proteins eIF2α, PERK, and IRE1α, thereby effectively alleviating clathrin-induced apoptosis in rat thylakoid cells (Wang et al., 2022). When AS-IV (50 μg/mL for 72 h) was administered to RMC cultured in high glucose, it downregulated miR-192 expression and inhibited high glucose-induced RMC overproliferation. In a rat DN model induced by STZ, AS-IV (40 mg/kg, for 8 weeks) also downregulated miR-192 expression and reduced the mRNA and protein levels of TGF-β1, Smad3, α-SMA, and COL-1, while increasing the mRNA and protein expression of Smad7. Therefore, AS-IV may treat DN by inhibiting interstitial hyperplasia and renal fibrosis through the TGF-β1/Smad/Mir-192 signaling pathway (Mao et al., 2019).

### 4.2 Podocytes

Podocytes represent a specialized subtype of differentiated epithelial cells that are responsible for upholding the structural and functional integrity of the glomerular filtration barrier, and furthermore determine the size of filtration proteins. When podocytes undergo apoptosis and are consequently lost, the filtration barrier structure collapses, which in turn leads to proteinuria (Lin et al., 2020; Wang et al., 2020).

The nephrotic syndrome refers to a urinary protein production caused by damage to podocytes, whereby the podocyte septum gap (known as the slit diaphragm or SD) and skeletal structure serve as essential components for maintaining filtration function. Following SHT (2.5, 5 and 10 g/kg for 12 weeks) intervention in a rat model of adriamycin nephropathy, the protein expression and RNA levels of SD-related proteins, namely, podocin and nephrin, were significantly upregulated in kidney tissues. Additionally, the protein expression and RNA levels of the podocyte skeleton-related protein CD2AP and the cytoskeletal damage component desmin were significantly decreased. These findings suggest that SHT exerts its mechanism of action on renal protection in rats with adriamycin nephropathy by improving glomerular podocyte lytic septum protein, maintaining podocyte skeleton homeostasis, and promoting glomerular podocyte repair after injury (Wang, 2016). AS-IV (6, 8, and 18 mg/kg for 2 weeks) improved renal function in db/db mice, reduced podocyte injury, and increased glomerular podocyte Klotho expression in glomerular foot cells (Xing et al., 2021). AS-II (at doses of 3.2 and 6.4 mg/kg for 9 weeks) improved STZ-induced proteinuria, renal histopathology, foot cell degeneration, and foot cell apoptosis in diabetic rats. This treatment partially restored the expression of renal mitochondrial dynamics-related and autophagy-related proteins, including Mfn2, Fis1, P62, and LC3. Moreover, AS-II increased the expression of PINK1 and Parkin, which are associated with mitochondrial autophagy, suggesting that AS-II may improve DN by regulating the Nrf2 and PINK1 pathways in diabetic rats (Gao et al., 2020; Su et al., 2021). AR was found to inhibit the expression of TGF-β1, FN, and MDA and increase SOD activity, TRPS expression, and the levels of α-dystroglycan and integrin to protect podocytes from damage (Wu and Li, 2016). Furthermore, AS-II downregulated TGF-β1, Smad3, and beta-catenin by inhibiting the expression of miR-21 and upregulating Smad7 expression to attenuate podocyte dedifferentiation (Cong, 2021). Additionally, AS-IV upregulated lncRNA-TUG1 and downregulated TRAF5 expression to reduce high glucose-induced podocyte injury (Lei et al., 2018). Xiao et al. investigated the effects of AS-IV on mouse podocytes and found that AS-IV promoted the connection between podocyte peduncles, improved their structural disorder, and alleviated glomerular basement membrane (GBM) thickening, while inhibiting excessive ECM. AS-IV downregulated TGF-β, N-cadherin, and α-SMA levels in podocytes and upregulated nephrin, E-cadherin levels, and SIRT1 expression. Furthermore, AS-IV reduced p65 acetylation levels and alleviated high glucose-induced podocyte EMT12 by enhancing autophagy, with the mechanism potentially acting through the regulation of the SIRT1-NF-κB pathway (Wang et al., 2019).

### 4.3 Glomerular capillary endothelial cells

Glomerular endothelial cells are intrinsic cells within the glomerulus and a crucial constituent of the nephron. They have the capacity to modulate glomerular filtration, playing a crucial role in the inner lining of the filtration barrier and are intricately involved in the pathophysiological processes of the glomeruli.

Administration of AR injection has been demonstrated to effectively prevent and repair STZ-induced vascular endothelial cell tight junction damage in diabetic rats, as well as reduce such injury in rats induced by high glucose peritoneal dialysis fluid (Shu et al., 2007). AR has been shown to decrease the release of endothelin, improve inflammation and oxidative stress in endothelial cells, balance intraglobular coagulation, and prevent apoptosis. This is accomplished through inhibiting the TNF-α/NF-κB signaling pathway, upregulating stem cell regulatory factors such as Nanog and Sox2, and inhibiting the expression of CAM, ROS, Fas, and MDA (Zhang and Que, 2020). Moreover, AR mono-AR injection has been found to upregulate the expression of sFlt-1 and downregulate VEGF, thereby promoting the proliferation and migration of glomerular endothelial cells (Cen et al., 2016). After treatment with AR combination (20 mL/kg for 12 weeks), unilateral nephrectomized and STZ-induced DN rats exhibited different biological indices in the glomeruli, including urine volume, blood glucose, glycosylated hemoglobin, lipids, VACM-1 and α-SMA. This treatment may protect renal endothelial cells by inhibiting VCAM-1 expression and preventing endothelial cell phenotypic transformation, thereby safeguarding renal tissue in DN rats (Zhang et al., 2014).

### 4.4 Renal tubular epithelial cells

Renal tubular epithelial cells represent the most fundamental structural units of renal tubules and play an essential role in the physiological function of the kidneys. Renal tubules are divided into proximal, distal, and fine segments, each with a unique epithelial cell function. Proximal renal tubular epithelial cells are responsible for reabsorbing glucose, amino acids, and proteins filtered from the glomerulus, as well as secreting carbonate ions to regulate the acid-base balance in the body. Distal renal tubular epithelial cells, on the other hand, mediate processes such as dilution, acidification, and concentration, resulting in a decrease in the specific gravity of urine. These cells also secrete hydrogen ions to regulate the acid-base balance in the body, thus maintaining homeostasis. Finally, the fine epithelial cells are the thinnest part of the renal tubule, and they are responsible for reabsorbing water, sodium, and urea. These cells are located between the proximal and distal tubules, are divided into descending and ascending branches, and can participate in the formation of the nephron medullary loop.

Injury to renal tubular epithelial cells is a major pathological process during ischemia-reperfusion injury (IRI) of the kidney. The localization and distribution of the sodium-potassium pump (Na^+^-K^+^-ATPase) within the renal tubular epithelium is a crucial factor affecting renal tubular function. In IRI, disruption of the Na^+^-K^+^-ATPase skeleton leads to a loss of epithelial cell function. SHT (1.5, 3.0 g/kg for 7 days) intervention after IRI resulted in an observed upregulation of Na^+^-K^+^-ATPase expression levels and improved distribution (Wei et al., 2013). Additionally, the sodium/dicarboxylic acid co-transporter protein (NaDC1), located primarily on the brush border side of the tubule, is a tubular polarity marker that acts in synergy with Na^+^-K^+^-ATPase to aid in the absorption of tricarboxylic acid cycle intermediates. Studies indicate that NaDC1 expression is decreased in renal tubular epithelial cells during IRI, resulting in a loss of tubular reabsorption function. A previous study indicated that intervention in a rat tubular epithelial cell hypoxia/reoxygenation model using serum containing SHT drug resulted in improved intracellular NaDC1 expression and distribution, suggesting that SHT could promote tubular reabsorption and repair tubular injury (Yang et al., 2014). AS-IV effectively inhibited UUO and TGF-β1-induced apoptosis in renal tubular epithelial cells by inhibiting Caspase-3 activation to prevent injury (Wang et al., 2014b). The anti-fibrotic mechanism of AS-IV prevents renal tubular epithelial cell apoptosis primarily through inhibition of Caspase-3 activation, and it may be mediated by downregulation of phosphorylated p38 and phosphorylated c-JunN-terminal kinase (Xu et al., 2014). Furthermore, APS inhibits the expression of MMP-2 and downregulates the expression of TIMP-1, TNF-α, and AngII (Lu and Wei, 2014; Sun et al., 2021). In addition, AS-IV inhibits multiple signaling pathways, including P38MAPK (Chen and Xie, 2019), TGF-β1/1-ILK (Zheng et al., 2021), and JAK/STAT (Guo et al., 2018). It also reduces the phosphorylation of P65 protein and effectively inhibits immune inflammation, apoptosis, and degradation of the extracellular matrix (ECM) in the renal tubular epithelium.

## 5 Discussion

SHT not only fortifies the spleen and augments the vital energy, nurtures the bodily fluids and invigorates the kidney to foster the fundamental vitality, but also remediates hemostasis and expels the toxins to alleviate the symptomatic manifestation. This precisely corresponds to the pathogenic mechanism of disease marked by the coexistence of deficiency and excess, ultimately culminating in the elimination of pathogenic factors and the restoration of the homeostasis of the organism. Within SHT, the combination of AR and *Atractylodis Macrocephalae Rhizoma* can invigorate the spleen and augment the Qi, with the added benefit of consolidating the surface and relieving fluid, whilst avoiding the accumulation of Qi stagnation. *Ligustri Lucidi Fructus* can both nourish the kidney and mollify the liver, as well as invigorate the Yin and enrich the blood, thus preventing blood depletion caused by blood-activating drugs. In addition, when paired with the appropriate dose of blood-activating and stasis-removing agents, it can gradually disperse stasis in individuals who present with such a condition, and further, for those who do not, it can still be utilized to enhance the effect of tonic drugs by removing the stagnation that such drugs may cause. Clinical practice has shown that SHT can improve the symptoms of Qi-Yin deficiency in patients with chronic nephritis and IgA(Chen et al., 2007). According to traditional Chinese medicine, IgAN is categorized as a disease involving blood in urine, edema, deficiency labor, and guangs. Its occurrence is due to the deficiency of positive and evil, where the positive cannot restrain the evil and win, resulting in a stalemate of positive and evil, ultimately leading to delayed and complicated diseases. The most common types of IgAN are liver and kidney yin deficiency, spleen and lung deficiency, qi and yin deficiency, spleen and kidney yang deficiency, among which the most commonly seen type is qi and yin deficiency.

**FIGURE 3:**
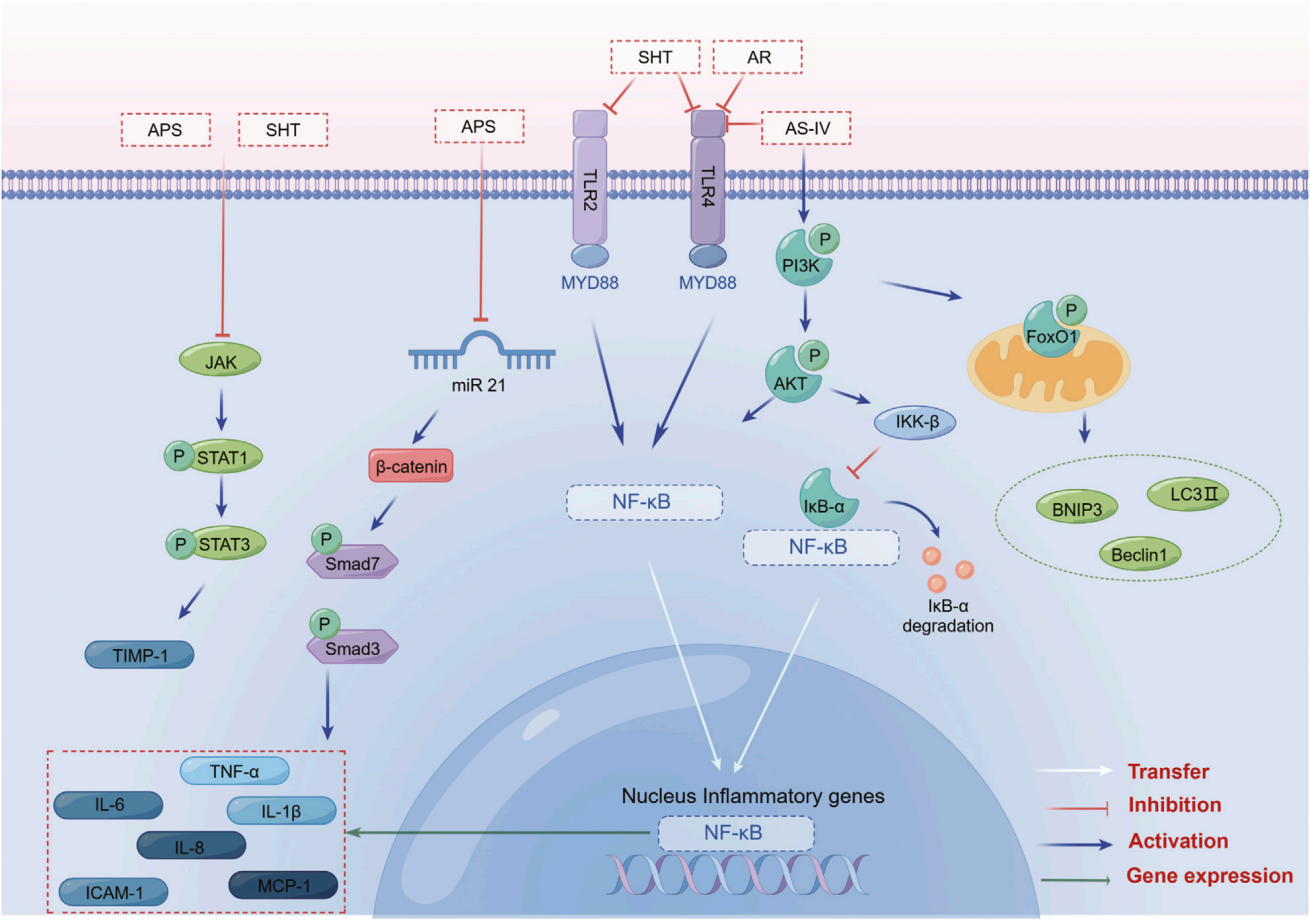
SHT, AR and AS-IV inhibit inflammation through the NF-κB, TLR, PI3K/AKT, Wnt/β-catenin, and JAK-STAT signaling pathways. Note: SHT, Fufang Shenhua tablet; AR, Astragali Radix; AS-IV, Astragaloside IV; APS: Astragalus polysaccharide. By Figdraw.

**FIGURE 4:**
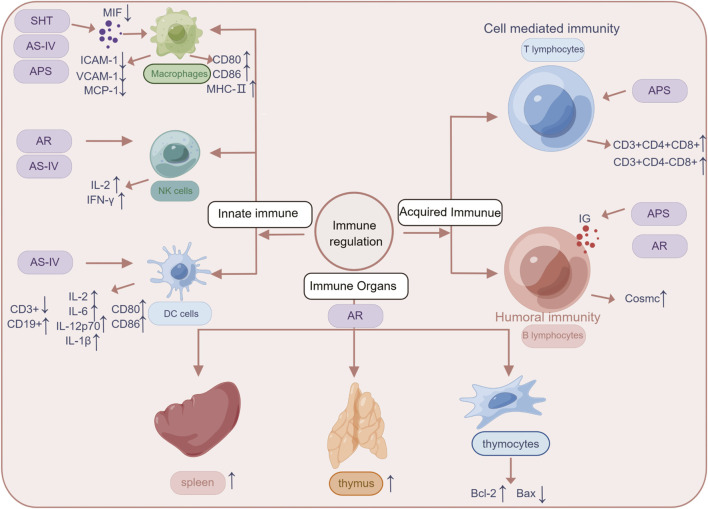
The mechanisms linked SHT, AR and AS-IV to the treatment of immune system diseases. Arrows and lines represent signal activation or inhibition. Note: SHT, Fufang Shenhua tablet; AR, Astragali Radix; AS-IV, Astragaloside IV; APS: Astragalus polysaccharide. By Figdraw.

The anti-inflammatory mechanism of SHT, AR and its active component AS-IV, is mainly linked to signaling pathways such as NF-κB, TLRs, PI3K/AKT, Wnt/β-catenin, and JAK-STAT (Figure 3). Furthermore, its immunomodulatory effects are vast, encompassing not only the intrinsic immune system but also the specific immune system by regulating immune organs, T lymphocytes, B lymphocytes, and various cytokines (Figure 4). NK cells play a vital role in anti-cancer and anti-immune diseases, and AR can promote the production of IL-2 and enhance the activity of NK cells, although its direct effects on NK cells themselves have yet to be fully investigated. Additionally, macrophages play a crucial role in the non-specific immune system, and existing AR studies have only focused on increasing the number and function of macrophages, without exploring in-depth the effects of AR on the immune regulatory patterns of macrophages.

**FIGURE 5:**
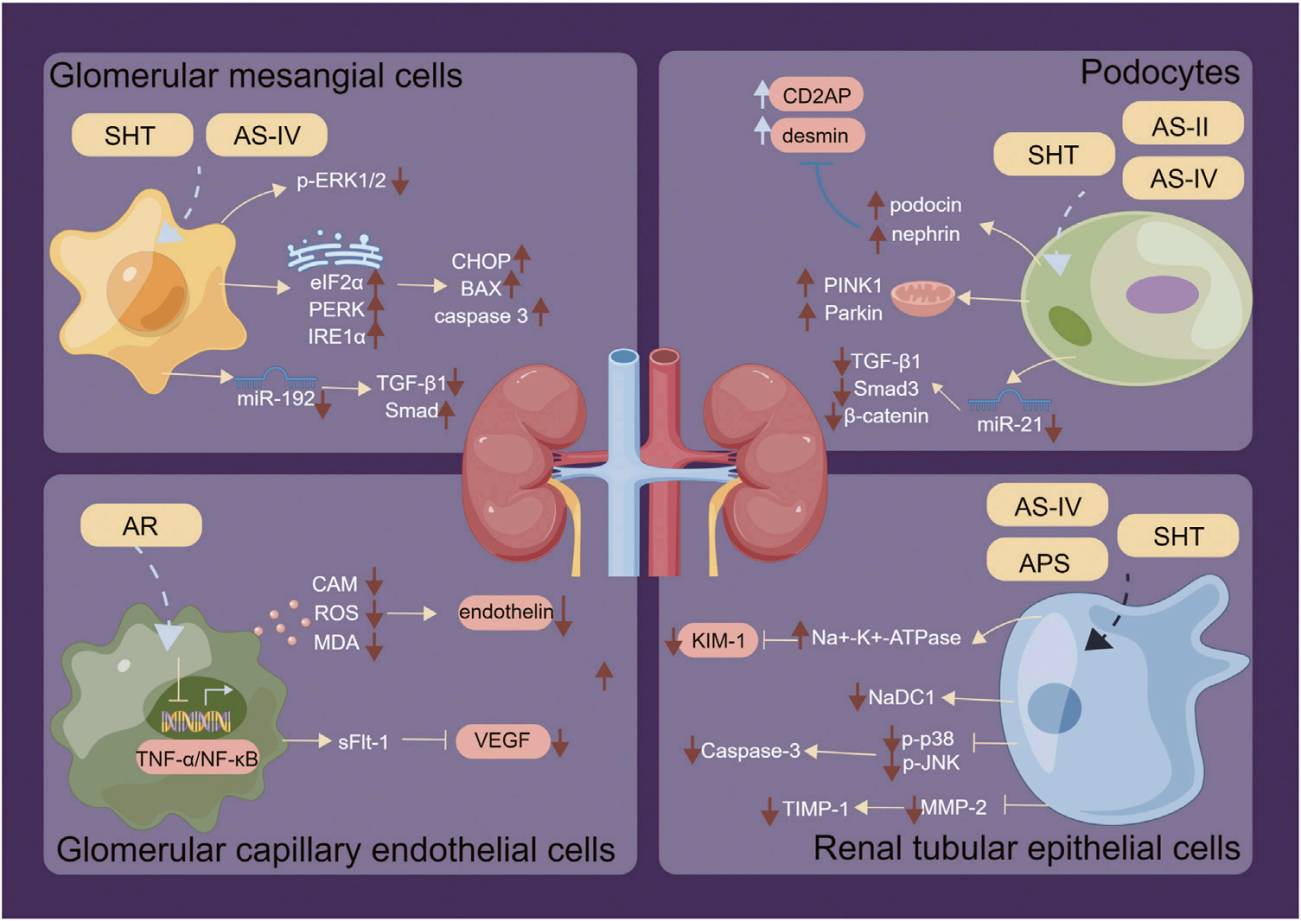
Regulatory mechanisms of SHT, AR and AS-IV on renal intrinsic cells. Arrows and lines represent signal activation or inhibition. Note: SHT, Fufang Shenhua tablet; AR, Astragali Radix; AS-IV, Astragaloside IV; APS: Astragalus polysaccharide; AS-II, Astragaloside II. By Figdraw.

Furthermore, SHT, AR, and AS-IV exhibit regulatory effects on renal intrinsic cells, such as glomerular interstitial cells, tubular epithelial cells, and podocytes (Figure 5). Although numerous basic studies have laid the groundwork for the clinical application of SHT (Table 2) and AR (Table 3) in the prevention and treatment of renal disease, the specific material basis for their efficacy remains unclear. The current studies have several limitations. Firstly, technical constraints prevent the precise identification of the exact composition of SHT and AR, either alone or in combination. Secondly, multi-component studies fail to reflect the true efficacy of these study drugs in renal disease. Thirdly, the efficacy of SHT and AR in treating renal disease has not been demonstrated by randomized controlled trials with large samples. Therefore, from a clinical perspective, the precise efficacy of SHT and AR in treating renal disease needs to be confirmed by more rigorous, randomized, and large-scale double-blind clinical trials. Non-etheless, more well-designed and properly conducted clinical trials are required before TCM can be utilized as a primary treatment for kidney disease. In addition to the general principles for ensuring the quality of clinical trials, such as multicenter collaboration, adequate sample size, randomized grouping, blinding, good clinical practice, and research ethics in human subjects, there are several aspects that need to be considered specifically when studying the clinical efficacy of TCM in the treatment of kidney disease. For TCM, the use of purified single active compounds or clarified mixtures of active ingredients may be more reliable, considering the advantages of multi-component and multi-target TCM. Quality control of the composition and stability of herbal medicines should always be performed during herbal medicine trials. Based on preclinical studies, appropriate doses and durations should be administered to the subjects.

**TABLE 2:** Molecular mechanisms responsible for the pharmacologic activity of SHT.

Model class	Intervention method	Molecular mechanism	References
RIRI in Wistar rats	SHT (1.5, 3.0 g/kg, p.o.) for 7 days	MyD88↓, TLR2↓, TLR4↓, TLRs, TNF-α↓, IL-6↓	Li et al. (2019)
MsPGN in Wistar rats	SHT (0.75, 1.5, 3 g/kg, p.o.) for 13 weeks	p-Erk1/2↓, cyclin D1↓, p21↑	Geng et al. (2016)
Adriamycin nephropathy in Wistar rats	SHT (2.5, 5,10 g/kg, p.o.) for 12 weeks	nephrin↑, podcin↑, CD2AP↓, desmin↓	Wang (2016)
RIRI in Wistar rats	SHT (1.5, 3.0 g/kg, p.o.) for 7 days	Na^+^-K^+^-ATPase↑, KIM-1↓	Yang et al. (2014)
RIRI in Wistar rats	SHT (1.5, 3.0 g/kg, p.o.) for 7 days	TLR2↓, TLR4↓, IL-6↓, IL-12↓, MyD88↓, IL-8↓, IFN-γ↓	(Zhen, 2013)
Anoxia/reoxygenation in HK-2	SHT (20.0 g/kg, p.o.) for 3 days	NaDC1	Wei et al. (2013)
RIRI in Wistar rats	SHT (1.5, 3.0 g/kg, p.o.) for 7 days	IL-8↓, IFN-γ↓, TLR2↓, TLR4↓	Zheng et al. (2012)
DN in SD rats	SHT (1.5 g/kg, p.o.) for 6 weeks	—	Geng et al. (2012)
CSSN in Wistar rats	SHT (45 g/kg, p.o.) for 4 weeks	Stat3↓, TIMP-1↓	Zhang et al. (2005)
5/6Nx in Wistar rats	SHT (2, 1, 0.5 g/kg, p.o.) for 12 weeks	—	Li et al. (2005)
5/6Nx in Wistar rats	SHT (5 g/kg, p.o.) for 12 weeks	MIF↓, ED-1↓	Li et al. (2005)
5/6Nx in Wistar rats	SHT (2, 1, 0.5 g/kg, p.o.) for 12 weeks	MIF↓, ED-1↓, PCNA↓, α-SMA↓, TGF-β1↓, FGF-2↓, CTGF↓, COL-1↓	Li (2005)
CSSN in Wistar rats	SHT (45 g/kg, p.o.) for 4 weeks	PCNA↓	Zhang et al. (2005)
CSSN in Wistar rats	SHT (45 g/kg, p.o.) for 4 weeks	Stat3↓, TIMP-1↓, ED-1↓, α-SMA↓	Zhang et al. (2005)

**TABLE 3:** The immune-modulatory and anti-inflammatory mechanisms of AR and its active constituents, and their impacts on intrinsic renal cells.

Active ingredient	Model class	Intervention method	Molecular mechanism	References
Huangqi Decoction	LPS-induced DN in SD rats/HG in RMCs	HD (1.5, 3.0 g/kg, p.o.) for 8 weeks	TLR4↓, p-IKK↓, p-NF-κB↓, IL-6↓, TNF-α↓, IL-1β↓	Li (2019)
AR injection	HG in HK-2	AR injection (2, 20, 200 mg/L) for 24, 48, 72 h	NF-κB↓	Mei et al. (2016)
AR injection	Immune complex glomerulonephritis models in Wistar rats	AR Injection (1 mL) for 7 weeks	NF-κB↓, IL-6↓, IL-2↓	Wang et al. (2001)
AR	Experimental lgA nephropathy in SD rats	AR (3 g/kg p.o.) for 6 weeks	p-NF-κBp65↓, MCP-1↓	Zhang et al. (2008)
AS-IV	UUO in C54BL6 mice	AS-IV (20, 40 mg/kg, p.o.) for 1 week	COL-IV↓, FN↓, CD68↓, CD3↓, TLR4↓, P65↓, IκBα↑,TNF-α↓, IL-1β↓	Zhou et al. (2017)
AR	STZ-induced DN in GK rats	AR (5 g/kg p.o.) for 8 weeks	p-PI3K↓, p-Akt↓, NF-κB↓, MCP-1↓	Shen (2016)
AS-IV	STZ-induced DN in SD rats	AS-IV (20, 40, 80 mg/kg, p.o.) for 8 weeks	p-PI3K↓, p-Akt↓, p-FoxO1↓, BNIP3↑, LC3Ⅱ↑, LC3Ⅰ↑, Beclin1↑	Ma et al. (2019)
AS-IV	UUO in C54BL6 mice	AS-IV (40 mg/kg, p.o.) for 8 weeks	miR-21↓, nephrin↑, α-SMA↓, β-catenin↓, TGF-β1↓, P-Smad3↓, and Smad7↑,Col IV↓, FN↓	Wang et al. (2018)
AS-IV	UUO in SD rats	AS-IV (3.3, 10, 33 mg/kg, p.o.) for 14 days before operation	Wnt3↓, Wnt4↓, Frizzled4↓, p-LRP5↓, p-LRP6↓, dishheveled↓, β-catenin↓, LEF-1↓, TCF-1↓, Snail↓, Jagged 1↓, Twist↓, MMP2↓, MMP7↓, PC↑, CK1↑, E-cadherin↑	Wang et al. (2014a)
AR	STZ-induced DN in Wistar rats	AR (2.1 g/kg p.o.) for 12 weeks	Wnt4↓, β-catenin↓,TGF-β1↓	Deng and Fang (2012)
APS	HG in HK-2	APS (200 mg/L) for 48 h	p-STAT1↓, p-STAT3↓, E-cadherin↓, α-SMA↓	Guo et al. (2018)
APS	RAW264.7	APS(0, 0.1, 1, 10, 50, 100 μg/mL) for 12 h	CD80↑, CD86↑, MHC-II↑	Luo et al. (2022)
AS-IV	Cyclophosphamide-induced in ICR mice	AS-IV (50 mg/kg p.o.) for 28 days	IL-2↑, IFN-γ↑	Hu et al. (2021)
iso-AS-IV	Cyclophosphamide-induced in ICR mice	iso-AS-IV (10, 20, 40 mg/kg p.o.) for 28 days	IL-6↑, IL-2↑, DC↑, CD3+↓, CD19+↑	Ruan et al. (2021)
Ast	Ovalbumin (OV A)-specific OT-II TCR Transgenic mice	Ast (28.2 ± 1.2 mg/L) Ast-Gal (38,800 ± 2.8 mg/L)	CD80↑, CD86↑, MHC-II↑, IL-1β↑, IL-6↑, IL-12↑	Li (2019)
AR	Deficiency of spleen qi in SD rats	AR (18.9, 12.6, 6.3 g/kg) for 15 days	Spleen index↑, thymus index↑	Zhang et al. (2021)
AR injection	Severe pneumonia in SD rats	AR (2.5, 5, 10 mL/kg) for 6 days	cleaved-caspase-3↓, Bax↓, Bcl-2↑	Lei et al. (2011)
APS	White Roman chickens	APS (1.0, 2.0, 4.0 mg/mL) for 48 h	IFN-γ↑, IL-2↑, IL-4↑, IL-10↑	Fan et al. (2012)
AS-IV	Splenic T and B cells, mouse peritoneal macrophages form BALB/c and C3H/HeJ mice	30, 100, and 150 μg/mL	B cells↑, macrophages↑, TLR4	Shao et al. (2004)
AR Injection	Peripheral B lymphocytes of IgAN patients200, 1,000 mg/ML		COSMc↓, IgA1↑	Ji et al. (2014)
APS	Splenic DCs, CD11chighCD45RBlow DCs CD11clowCD45RBhigh DCs, splenic CD4^+^ T cells of male BALB/c mice	50, 100, and 200 μg/mL	shifting of Th2 to Th1↑, T cells↑	Liu et al. (2011)
AS-IV	Tunicamycin Induced Mesangial Cells Apoptosis	50 μg/mL	p-eIF2α↓, p-PERK↓, p-IREα↓, CHOP↓, BAX↓, Cleaved caspase 3↓	Wang et al. (2022)
AS-IV	STZ-induced DN in SD rats/HG in RMCs	AS-IV (40 mg/kg, p.o.) for 8 weeks	TGF-β1↓, Smad7↓, miR-192↓, Smad7↓,TGF-β1↓, α-SMA↓,COL-1↓	Mao et al. (2019)
AS-IV	db/db mice	AS-IV (6,8,18 mg/kg, p.o.) for 2 weeks	PPARγ↑, FoxO1↑, Klotho↑	Xing et al. (2021)
AS-II	STZ-induced DN in SD rats	AS-II (3.2, 6.4 mg/kg, p.o.) for 9 weeks	Mfn2↑, Fis1↑, P62↑, LC↑3, PINK1↑, Parkin↑	Su et al. (2021)
AS-II	STZ-induced DN in SD rats	AS-II (3.2 mg/kg, p.o.) for 9 weeks	Nephrin↑, WT1↑, caspase-3↓	Gao et al. (2020)
AS-IV	STZ-induced DN in SD rats	AS-II (5 mg/kg, p.o.) for 12 weeks	TRAF5↓, TUG1↑	Lei et al. (2018)
Huangqi Weimao mixture	STZ-induced DN in SD rats	20 mL/kg for 12 weeks	VACM-1↓, α-SMA↓	Zhang et al. (2014)
AS-IV	UUO in SD rats	AS-IV (3.3, 10, 33 mg/kg, p.o.) for 14 days before operation	TGF-β↓, CTGF↓, α-SMA↓, p-Smad3/2↓,COL-I↓, COL-III↓	Wang et al. (2014b)
AS-IV	UUO in C54BL6 mice	AS-IV (20 mg/kg, i.p.) for 7 days	cleaved-caspase-3↓, p-p38↓, p-JNK↓	Xu et al. (2014)
APS	UUO in SD rats	APS (0.5,1 g/kg p.o.) for 21 days	MMP-2↑, TGF-β1↓, TIMP-1↓, AngⅡ↓	Lu and Wei (2014)
APS	Sepsis-Induced AKI in C57BL/6 mice	APS (1,3,5 mg/kg, i.p.) for 3 days before operation	TNF-α↓, IL-1β↓, IL-6↓, IL-8↓, caspase-3↓, caspase-9↓, Bax↓, Bcl-2↑, α-SMΑ↓, Vimentin↓	Sun et al. (2021)
APS	Hypoxia reoxygenation in HK2	100, 200, 300 mg/L	IL-1β↓, TNF-α↓, p-p38MAPK↓	Chen and Xie (2019)
APS	hypertensive mice	200 mg/kg	IL-1β↓, IL-6↓, α-SMA↓, collagen I↓, collagen III↓	Liu et al. (2011)

To better understand the pharmacological effects of SHT and AR components, techniques such as high performance liquid chromatography and mass spectrometry should be employed to identify drug components and provide a clearer material basis for pharmacological studies. Future studies should not only focus on the phenotypes of the drugs, but also on further *in vitro* mechanism studies to better elucidate their pharmacological effects. However, it is important to note that more objective, scientific approaches and long-term studies are needed to demonstrate the safety and efficacy of SHT and AR in the treatment of renal diseases, which are complex and involve multiple factors. While the AR in SHT has shown promise in the treatment of renal diseases, further studies on the mechanisms of action and preparation of compounds with active ingredients of SHT and AR are needed. Additionally, definitive quality markers (Q-Markers) must be identified to provide a more scientific basis for future drug production and to more clearly define the targets for the treatment of renal diseases.

In conclusion, SHT, AR and its active component AS-IV exhibit anti-inflammatory and immunomodulatory effects through various pathways and mechanisms. Nevertheless, a more comprehensive and profound comprehension of its targets and the paucity of studies on adverse effects require further pharmacological and clinical experimental investigations. These studies will help identify therapeutic mechanisms and targets, as well as provide a dependable theoretical foundation for clinical treatment.
